# Frequency of Vitamin B12 Deficiency in Type 2 Diabetic Patients Taking Metformin

**DOI:** 10.7759/cureus.22924

**Published:** 2022-03-07

**Authors:** Fraz Ahmed Baig, Saad Khan, Amber Rizwan

**Affiliations:** 1 Internal Medicine, Nottingham University Hospitals, Nottingham, GBR; 2 Internal Medicine, James Cook University Hospital, South Tees Hospitals NHS Foundation Trust, Middlesbrough, GBR; 3 Family Medicine, Jinnah Post Graduate Medical Center, Karachi, PAK

**Keywords:** : metformin, diabetes mellitus type 2, functional vitamin b12 deficiency, vitamin b12 supplementation, vitamin b12

## Abstract

Introduction

Type 2 diabetes mellitus (DM) is a prevalent global health problem and is on a constant rise, especially in middle- and low-income countries. Vitamin B12 malabsorption is one of the reported side effects of metformin. Our study aims to assess the correlation of B12 deficiency in type 2 diabetics using metformin for their treatment.

Methods

This case-control study was conducted in a tertiary care hospital in Pakistan from February 2021 to December 2021. Patients (n=100) with a documented diagnosis of type 2 DM on metformin monotherapy for a minimum of six months were enrolled via consecutive convenient non-probability sampling. Another 100 patients without a history of diabetes were included in the study as a control group for comparison.

Results

Serum vitamin B-12 levels were higher in the non-diabetic participants as compared to the diabetic group (301.71 ± 72.12 vs. 189.25 ± 31.22; p-value: <0.0001). Hypovitaminosis was more significant in the diabetic group (p-value: 0.0000). Serum vitamin B12 levels were found to be declining with the increasing duration of metformin use (p-value: <0.0001).

Conclusion

Our study found a significant effect of vitamin B12 deficiency in metformin-treated patients. Therefore, it is prudent to recognize B12 deficiency as a potential side effect of long-term use of metformin. A periodic screening of B12 in such patients and subsequent supplementation of vitamin B12 is an effective and safe means of prevention of development or worsening of peripheral nerve damage and other clinical manifestations.

## Introduction

Type 2 diabetes mellitus (T2DM) is a prevalent global health problem and is on a constant rise, especially in middle- and low-income countries [1]. Its predominance in Pakistan ranges from 7.2% to 19.21% [2]. Metformin is the drug of choice in the majority of type 2 DM patients. Depending upon the characteristics of each patient, other alternatives or second-line treatment options for type 2 DM are tailored for each individual [3].

The most frequent side effects related to metformin are not severe and usually result in temporary and self-restricting gastrointestinal (GI) disturbance [4]. Apart from GI disturbances, other clinically important side effects reported include vitamin B12 malabsorption [5]. A Brazilian study on patients receiving extended treatment with metformin concluded that the frequency of vitamin B12 deficiency ranges from 5.8% to 30% [5].

Despite a high prevalence of T2DM in Pakistan, extremely restricted information regarding the side effects of metformin is available, especially in terms of vitamin B12 deficiency in patients using metformin. Our study aims to assess the correlation of B12 deficiency in type 2 diabetics using metformin for their treatment.

## Materials and methods

This case-control study was conducted in a tertiary care hospital in Pakistan from February 2021 to December 2021. Patients were enrolled via consecutive convenient non-probability sampling. Inclusion criteria for this study were patients with a documented diagnosis of type 2 DM between the ages of 20 to 60 years, of either gender, who were on metformin monotherapy for a minimum of six months. Pregnant, lactating women, vegans, and participants with diagnosed diabetic peripheral neuropathy were excluded from the study. The sample size was calculated by using statistical software OpenEpi (www.OpenEpi.com) with 80% power of the test and 95% confidence interval. Considering the 5.8% frequency of B12 deficiency in metformin users [5], the sample size calculated was 84. Considering dropping out, 100 patients were taken in the study. Another 100 patients without a history of diabetes were included in the study as a control group for comparison.

All the patients meeting the above-mentioned inclusion criteria were enrolled from the outpatient department of internal medicine after taking approval from the ethical review board of Jinnah Sindh Medical University (JSMU/IRB/2021-28C). The protocol risk was explained to the patient in detail. Informed consent was taken before each procedure and patients’ privacy and confidentiality were entertained. All the data was encrypted. Patients’ demographics and other relevant information were recorded in self-structured questionnaires.

After registration, participants' venous blood was taken via cubital blood and sent to the laboratory for vitamin B12 assay. All labs were taken in the morning to avoid the risk of diurnal variation. Vitamin B12 levels were measured by competitive-binding immunoenzymatic assays using a Unicel DxI 800 analyzer (Beckman Coulter, Inc., Brea, USA). Participants were labeled as having vitamin B12 deficiency if their serum levels were less than 200 pg/mL [6].

All the data obtained was analyzed in Statistical Package for Social Science (SPSS), version 20 or above (IBM Corp., Armonk, USA). Mean and standard deviation were calculated for the age and serum B12 values. Frequency and percentages were calculated for the gender and frequency of vitamin B12-deficient participants. The mean age was compared using independent t-tests, while gender and other characteristics were compared using the Chi-square test. A p-value of less than 0.05 was considered significant.

## Results

No statistically significant difference between the characteristics of the diabetic and non-diabetic groups was found upon age (p-value: 0.4330) and gender (p-value: 0.7718) comparison. Other factors including smoking, hypertension, and chronic kidney disease are also compared as follows (Table 1).

**Table 1:** Demographics and risk factors of participants CKD: chronic kidney disease, HTN: hypertension

Characteristics	Diabetes group (n=100)	Non-diabetes group (n=100)	p-value
Age (in years)	48 ± 09	47 ± 09	0.4330
Gender (%)			
Male	62	60	0.7718
Female	38	40	
Other factors (%)			
Smoking	22	24	0.7368
HTN	31	27	0.5330
CKD	09	07	0.6021

Serum vitamin B12 levels were higher in the non-diabetic participants as compared to the diabetic group (301.71 ± 72.12 vs. 189.25 ± 31.22; p-value: <0.0001). Hypovitaminosis was more significant in the diabetic group (p-value: 0.0000) (Table 2).

**Table 2:** Comparison of vitamin B12 levels between the diabetic and non-diabetic groups pg/mL: picograms per milliliter

Characteristics	Diabetes group (n=100)	Non-diabetes group (n=100)	p-value
Serum B-12 levels (pg/mL)	189.25 ± 31.22	301.71 ± 72.12	<0.0001
Hypovitaminosis (%)	39	12	0.0000

Serum vitamin B12 levels were found to be declining with the increasing duration of metformin use (p-value: <0.0001) (Table 3).

**Table 3:** Correlation of duration of metformin use with serum B-12 levels pg/mL: picograms per milliliter

Duration of metformin use	Serum B-12 levels (pg/mL)	p-value
Less than 1 year (n=12)	231.82 ± 51.02	<0.0001
Between 1 to 2 years (n=51)	186.21 ± 28.34	
More than 2 years (n=37)	161.61 ± 25.12	

## Discussion

Our study demonstrated that serum vitamin B12 levels were significantly lower in diabetics (189.25 ± 31.22) compared to non-diabetics (301.71 ± 72.12). Moreover, hypovitaminosis was considerably observed more in the non-diabetic group. The association between low vitamin B12 levels and metformin has been supported by numerous studies [7-9]. However, some contradictory studies state that there is no evidence providing the association between the two [10,11]. Upon further investigation, our study proved that with increasing duration of time, vitamin B12 levels were seen to drop. This finding has been anchored by high-level studies [9,12,13], due to which the 2017 American Diabetes Association treatment guidelines include keeping a regular check on vitamin B12 levels in diabetic patients using metformin [14]. 

The most possible explanation proposed for the role of metformin in lowering vitamin B12 levels is that it hinders the smooth functioning of the calcium-regulated membrane that helps in the absorption of the vitamin B12-intrinsic factor complex in the ileum [15]. This led to investigating whether the administration of multivitamin supplementation could help avoid this deficiency since minute quantities of vitamin B12 are present in supplements (2 to 30 ug/d). This was assessed in a retrospective study; it concluded that intake of vitamin supplements in patients taking metformin was seen to report fewer events of vitamin B12 deficiency (adjusted OR=0.14; 95% CI=0.04, 0.54) compared to the group that did not take multivitamins [16].

Our study bears a few limitations. We only assessed total vitamin B12 levels; however, holotranscobalamin II or methylmalonic acid could have been monitored since they accurately express vitamin B12 levels. Moreover, we did note the diet of the participants. Consequently, we could not rule out the possibility of other factors in significantly lowering vitamin B12. This points towards the fact that future studies should be conducted with a larger sample size to confirm the findings of our study.

## Conclusions

Considering the rising prevalence of type 2 DM and the favorable safety and efficacy profile of metformin, its use as an insulin-sensitizing agent is widespread. Our study found a significant correlation between vitamin B12 deficiency in metformin-treated patients. Therefore, it is prudent to recognize B12 deficiency as a potential side effect of long-term use of metformin. A periodic screening of B12 in such patients and subsequent supplementation of vitamin B12 is an effective and safe means of prevention of development or worsening of peripheral nerve damage and other clinical manifestations.
